# Early Cervical Cancer: Predictive Relevance of Preoperative 3-Tesla Multiparametric Magnetic Resonance Imaging

**DOI:** 10.1155/2018/9120753

**Published:** 2018-08-01

**Authors:** Hyun Jin Roh, Kyung Bin Kim, Jong Hwa Lee, Hwa Jung Kim, Yong-Soon Kwon, Sang Hun Lee

**Affiliations:** ^1^Department of Obstetrics and Gynecology, University of Ulsan College of Medicine, Ulsan University Hospital, Ulsan, Republic of Korea; ^2^Department of Pathology, University of Ulsan College of Medicine, Ulsan University Hospital, Ulsan, Republic of Korea; ^3^Department of Radiology, University of Ulsan College of Medicine, Ulsan University Hospital, Ulsan, Republic of Korea; ^4^Department of Clinical Epidemiology and Biostatics, University of Ulsan College of Medicine, Asan Medical Center, Seoul, Republic of Korea

## Abstract

**Objective:**

We assess the predictive significance of preoperative 3-Tesla multiparametric MRI findings.

**Methods:**

A total of 260 patients with FIGO IA2-IIA cervical cancer underwent primary surgical treatment between 2007 and 2016. Univariable and multivariable logistic regression analyses were used to assess the incremental prognostic significance.

**Results:**

The clinical predictive factors associated with pT2b disease were MRI parametrial invasion (PMI) (adjusted odds ratio (AOR) 3.77, 95% confidence interval(CI) 1.62-8.79; P=0.02) and MRI uterine corpus invasion (UCI) (AOR 9.99, 95% CI 4.11-24.32; P<0.0001). In multivariable analysis, for underdiagnoses, histologically squamous carcinoma versus adenocarcinoma and adenosquamous carcinoma (AOR 2.07, 95% CI 1.06-4.07; P=0.034) and MRI tumor size (AOR 0.76, 95% CI 0.63-0.92; P=0.005) were significant predictors; for overdiagnoses, these results were MRI tumor size (AOR 1.51, 95% CI 1.06-2.16; P=0.023), MRI PMI (AOR 71.73, 95% CI 8.89-611.38; P<0.0001) and MRI UCI (AOR 0.19, 95% CI 0.01-1.01; P=0.051).

**Conclusion:**

PMI and UCI on T2-weighted images through preoperative 3T MRI are useful coefficients for accurate prediction of the pT2b stage; however, careful surveillance is required. Therefore, preoperative decision-making for early cervical cancer patients based on MRI diagnosis should be considered carefully, particularly in the presence of factors that are known to increase the likelihood of misdiagnosis.

## 1. Introduction

Currently, the International Federation of Gynecology and Obstetrics (FIGO) classification is the most commonly used method to determine the clinical stage of cervical cancer. The FIGO staging guidelines were most recently updated in 2009 by the FIGO Committee on Gynecologic Oncology [1]. Stage 0 is no longer included in the FIGO 2009 staging [1]. The clinical FIGO classification is exclusively used because cancer staging is a generally accepted universal stratification system for communication purposes among institutions. Despite continuous studies for the accurate staging of cervical cancer, the reality is that no entirely accurate method exists at present. Regarding the limitations of the clinical FIGO classification as the most commonly used method, the overall error rate of clinical staging for cervical cancer compared with surgical staging is between 20 and 66%. Clinical staging has revealed more inaccurate diagnoses, especially in advanced stages [2–6]. Furthermore, when distinguishing between stages IB and IIB, tumor invasion of the parametrium is one of the most important considerations. This determination completely depends on the attending gynecologist's findings via manual palpation, which is intrinsically subjective [7]. As complementary measures, the National Comprehensive Cancer Network (NCCN) suggests the use of imaging methods such as CT, MRI, and combined PET-CT in guiding treatment options and individual treatment design, but this practice guideline is not generally accepted for formal and official staging purposes. Accurate staging of cervical cancer is essential when making the therapeutic decision between radiation and surgery [8, 9]. For early-stage disease, such as stage I and selected IIA, surgery or radiation therapy can be applied as treatment options [8, 9]. There are various treatments for voluminous stage IB according to the location and size of the tumor. For advanced stages (stage IIB and greater), radiation therapy is typically chosen [8–13]. Thus, various methods for the accurate prognostic detection of parametrial invasion have been proposed [14–20]. However, more information is required to formulate accurate decisions regarding treatment for individual patients. With the introduction of more sophisticated MRIs, such as “multiparametric” imaging (the combination or morphologic and functional MRI sequences, including T2-weighted (T2W), diffusion-weighted (DW), and magnetic resonance spectroscopic imaging and higher field-strength capabilities (3T versus 1.5T MRI)), an improvement in staging cervical cancer is expected. A few studies have reported that the accuracy of PMI by MRI is associated with the primary tumor size and uterine corpus invasion in patients with clinically localized cervical cancer who underwent radical hysterectomy (RH) [14–16, 18–21].

Therefore, the present study aimed to optimize the predictability of staging for cervical cancer by analyzing the incremental predictive significance of preoperative 3-Tesla (3T) multiparametric MRI findings for predicting pathologic T2b and predictive factors associated with MRI stage misdiagnosis in patients with early cervical cancer underwent radical hysterectomy.

## 2. Methods and Methods

### 2.1. Patient Selection and Treatment

The cohort in this study included 260 documented cervical cancer patients with clinical FIGO stages IA2 and IIA who underwent primary surgical treatment between January 2007 and December 2016 following a 3T MP MRI examination at Ulsan University Hospital.

Patient age ranged from 24 to 75 with a mean value of 49.3 years. Tissue diagnosis of cervical cancer was performed through biopsy specimens for all patients. Clinical and MR imaging data were recorded retrospectively based on the patients' medical records and PACs system by one author.

Inclusion criteria were (a) biopsy-documented invasive cervical cancer by a loop electrosurgical excision procedure (LEEP) or a cone biopsy or punch biopsy; at least 7 days after a biopsy, MRI was performed to prevent false-positive findings due to biopsy inflammation, (b) clinical FIGO stage IA, IB, or IIA, (c) histology of squamous cell carcinoma or adenocarcinoma or adenosquamous carcinoma, (d) no medical or surgical contraindications to radical hysterectomy with pelvic lymph node dissection (PLND) with or without para-aortic lymph node sampling (PALS) and dissection (PLND), (f) having an Eastern Cooperative Oncology Group (ECOG) performance status of 0-1, and (g) provided informed consent.

Patients with the following criteria were excluded: (a) previously treated with radiotherapy and/or chemotherapy for cervical cancer, (b) contra-indication to the MRI procedure including patients unwilling to go through contrast-enhanced MRI, (c) a previous diagnosis of vulva and vaginal cancer, and (d) concomitant pregnancy.

### 2.2. Conventional Staging Work-Up

FIGO staging was determined based on findings from physical examination, such as bimanual pelvic examination, endoscopic studies (cystoscopy and sigmoidoscopy), and radiologic studies (chest radiography, intravenous pyelogram, and barium enema) after histologic confirmation of invasive cervical cancer.

### 2.3. Surgical Technique

All 260 patients underwent surgery. Regarding the surgical procedure types, hysterectomy (laparoscopic, transabdominal, or transvaginal), radical hysterectomy (laparoscopic, transabdominal, robotic), and radical trachelectomy for fertility preservation were permitted. All patients underwent pelvic lymph node sampling or dissection. For patients with large tumors (> 2 cm), para-aortic lymph node sampling was performed.

### 2.4. MRI Scanning

#### 2.4.1. MRI Protocol

All MRI examinations were scanned on a 3T whole-body MRI scanner (Intera Achieva 3T, Philips Medical System, Best, the Netherlands) using phased-array techniques involving pelvic or torso phased-array coils. Multiparametric imaging sequence parameters (Table S1 in the Supplementary Appendix) included multiplanar T2W, T1-weighted (T1W), and DW imaging with multiple b value (0-800 s/mm2) of the cervix. Gadopentetate dimeglumine contrast (Magnevist; Schering, Berlin, and Germany) in all the patients was administered intravenously at a weight-based dosing of 0.2 ml/kg with a bolus injection rate of 2 ml/sec using an automatic injector, which was followed by a 20-ml saline bolus injection.

#### 2.4.2. MRI Interpretation

All MR images were assessed retrospectively based on all the available clinical data by a gynecological radiologist with more than 10 years of experience in the field of gynecologic cancer imaging. A gynecological radiologist described each MRI finding including the presence of primary tumor; depth of stromal invasion (no invasion, partial invasion, and complete invasion); extension to uterine corpus, vagina, parametrium, pelvic sidewall, urinary bladder, and rectum; and pelvic and para-aortic lymph node metastasis. At the end of the imaging evaluation session, multiparametric MRI staging was determined by MRI T category using both FIGO and TNM stratification criteria (i.e., AJCC-TNM Cancer Staging System, 7th edition) [22, 23] as shown in Table S2. When no tumor was identified by MR imaging despite findings of malignant cells on biopsy, a radiologic stage of MRI-invisible IB1 was assigned in previous study. However, in this study, MRI-invisible IB1 was independently classified as MRI-invisible-T0 stage (Figures 4 and 5) rather than included in the MRI IB1 stage.

#### 2.4.3. Histopathologic Analysis

The procedure was performed and the surgical specimens of the 260 radical hysterectomy and trachelectomy patients were prepared according to standard methods.

All surgical specimens were fixed in formalin and embedded in paraffin. A dedicated and experienced gynecologic oncology pathologist reviewed all of the H&E-stained sections for all patients. The overall stage was determined according to both FIGO and TNM stratification criteria. The TNM classification based on the American Joint Committee on Cancer (AJCC) staging system, 7th edition (2010), was the radical hysterectomy T category [23].

In addition to pTNM classification based on AJCC staging manual, if no tumor was found in the cervix in the surgical specimen after positive biopsy for infiltrating carcinoma, we stratified as pT0b1 stage, which was classified as a class A0 in Meigs J.V. et al. at surgical and pathologic classification of the uterine cervix [3].

### 2.5. Statistical Analysis

The distributions of the study cohort's clinical and pathologic characteristics were calculated and are presented in Table 1. The clinical predictive factors were analyzed by the distributions of categorical factors stratified by MRI T category (T0, T1b, T2a, and T2b).

Continuous variables were compared using ANOVA or the Kruskal-Wallis test, and categorical variables were compared using the *χ*2 test or Fisher's exact test.

Accuracy was calculated using the final surgical pathology examinations as the reference. A logistic regression multivariable analysis (MVA) (Tables 2 and 3) was used to assess the clinical factors for predicting upstaging to pT2b and the clinical factors associated with MRI stage misdiagnosis in FIGO IA-IIA cervical cancer patients underwent radical hysterectomy, adjusting for age, menopause, parity, and BMI, cesarean section, history of at least one normal delivery, menopause, SCC ag, grade, MRI stage, clinical FIGO stage, MRI tumor size, MRI Pelvic LN invasion, MRI parametrial invasion, MRI uterine corpus invasion, MRI deep stromal invasion, and MRI vaginal invasion.

Unadjusted and adjusted odds ratios (UORs and AORs, respectively) were calculated for each clinical covariate, and these values were reported with 95% confidence intervals (CIs). Area under the receiver operating characteristic (ROC) curve (A_Z_) was used to determine the cut-off lesion size measured on MRI for optimal accuracy to predict the effect of large tumor size on misdiagnosis (overdiagnosis and underdiagnosis). A P value <0.05 was considered statistically significant. IBM SPSS 21.0 statistics software was used (IBM Institute, Inc., Armonk, NY, USA).

## 3. Results

### 3.1. Description of the Study Cohort Stratified by MRI T Category

The clinical and pathologic characteristics of the 260 women in this study cohort are shown in Table 1 and categorized by MRI T category (T0, T1b, T2a, and T2b).

### 3.2. Performance Characteristics of 3T Multiparametric MRI

The overall accuracy of 3T multiparametric MRI for predicting parametrial invasion (PMI) was 83%. The sensitivity, specificity, positive predictive value (PPV), and negative predictive value (NPV) of 3T multiparametric MRI for predicting pathologic parametrial invasion were 62%, 88%, 53%, and 91%, respectively

### 3.3. Logistic Regression MVA: Pathologic T2b Disease Outcome

A subset of 203 women (excluding 57 with MRI T0 stage) was stratified according to the MRI finding. Based on a univariable analysis of these women that excluded those with MRI T0 stage, MRI PMI (AOR 3.77, 95% CI 1.62-8.79; P=0.002), and MRI uterine corpus invasion (UCI) (AOR 9.99, 95% CI 4.11-24.32; P<0.0001) were associated with increased odds of having pT2b disease after adjusting clinical predictive factors as shown in Table 2.

### 3.4. Logistic Regression MVA: Underdiagnosis and Overdiagnosis

The accuracy of MRI staging with regard to the histologic specimens from 260 cervical cancer patients with clinical FIGO stages IA-IIA was 69%, whereas the overall error rates varied from 15% for MRI T1b to 46% for MRI T2b. According to our findings, the MRI stage underdiagnosed 18% of patients and overdiagnosed 14%.

The underdiagnosis rates were 46%, 13%, and 20% for MRI stages T0, Tb1, and T2a, respectively. The overdiagnosis rates were 2%, 70%, and 46% for MRI stages Tb1, T2a and T2b, respectively (Tables 4, and 5). The factors influencing the accuracy of MRI staging were further investigated (Table 3).

In the underdiagnosis cases (Figures 6 and 7), the predictive factors included a histology of squamous versus adenocarcinoma and adenosquamous carcinoma (AOR 2.07, 95% CI 1.06-4,07; P=0.034) and MRI tumor size (AOR 0.76, 95% CI 0.63-0.92; P=0.005). In the overdiagnosis cases (Figures 2 and 3), the predictive factors included MRI tumor size (AOR 1.51, 95% CI 1.06-2.16; P=0.023), MRI uterine corpus invasion (AOR 0.13, 95% CI 0.03-0.49; P=0.003), and MRI parametrial invasion (AOR 73.73, 95% CI 8.89-611.38; P<0.0001) after preoperative predictors associated with MRI stage misdiagnosis were adjusted as shown in Table 3.

**Figure 1:**
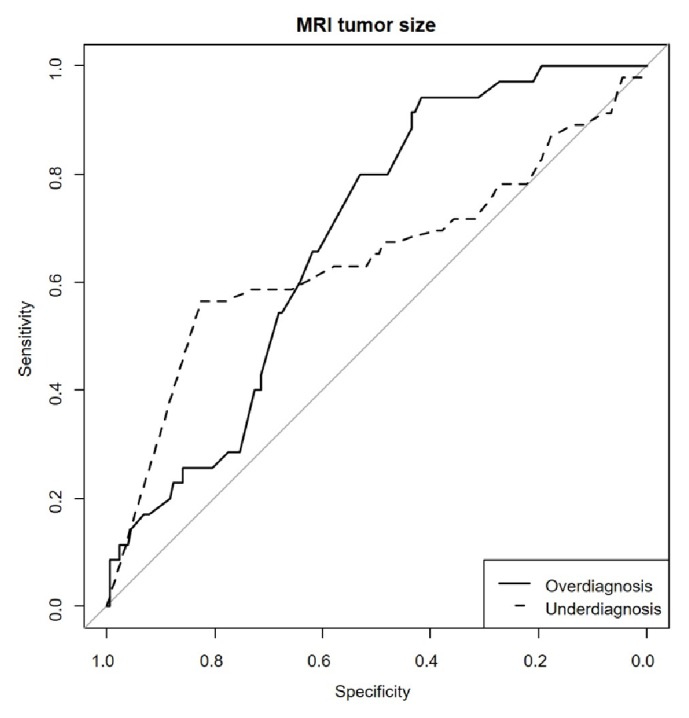
Comparison of finial pathologic stage versus multiparametric MRI radiologic stage versus clinical FIGO stage.

The area under receiver operating characteristics curve analysis of the cut-off lesion size measured on MRI for optimal accuracy to predict the effect of large tumor size on misdiagnosis (overdiagnosis and underdiagnosis) is shown in Figure 1.

With overdiagnosis, a tumor cut-off size of 2.9 cm (approximated on T2W images) was observed with 80% sensitivity and 53% specificity (AZ=0.68, 95% CI 0.60-0.77; Table 6), in underdiagnosis, a tumor cut-off size 0.25 cm (approximated on T2W images) was observed with 57% sensitivity and 83% specificity (AZ=0.64, 95% CI 0.54-0.75; Table 6)

## 4. Discussion

Previous studies have evaluated the ability of 1.5 T MRI to determine the presence of parametrial invasion and have considered whether the information from 1.5 T MRI might meaningfully supplement the known predictive factors, including primary tumor size, the depth of cervical stromal invasion, and extension to the uterine corpus in patients with cervical cancer patients [7, 15–20, 24, 25].

However, few studies using 1.5 T MRI have attempted to identify established factors associated with upstaging at the radical hysterectomy in patients with early cervical cancer IA-IIA. To determine an optimal treatment plan for patients scheduled to undergo radical hysterectomy, accurate risk assessment is necessary because the decision to use concurrent chemoradiation therapy with radical hysterectomy depends on the patient's clinicopathologic risk factors. The identification of high-risk factors is essential to avoid overtreatment, which carries substantial acute and chronic adverse effects [4, 26]. Therefore, we assessed the predictive relevance of preoperative 3T MRI findings in predicting pathologic stage T2b and the clinical factors associated with MRI stage misdiagnosis in patients with FIGO stages IA-IIA2 cervical cancer underwent radical hysterectomy.

In 2005, Hricak et al. reported a prospective 25-center clinical study of 172 patients. In the detection of advanced stage (≥IIB), the sensitivity, specificity, NPV, and PPV for MRI were 53%, 74%, 85%, and 37%, respectively. In our study, the sensitivity, specificity, NPV, and PPV of 3T multiparametric MRI to predict pathologic parametrial invasion were 62%, 88%, 91%, and 53%, respectively. Our results for 3T multiparametric MRI are higher than those reported in prior studies [17].

Hicak et al. [21] indicated a strong correlation between parametrial invasion and uterine corpus invasion and Peter de Boer's systemic review [20] noted a high accuracy rate for MRI in detecting cancer involvement of the uterine internal os in cervical cancer. This study confirmed the results of those two studies of MRI parametrial invasion and MRI uterine corpus invasion as credible predictive factors for predicting pT2b.

It is noteworthy that radiologists have been providing high specificity readings to prevent unnecessary abolition of curative surgery while favoring external beam radiotherapy over RH in cases of suspected pT2b cervical cancer, whereas clinicians are more likely concerned with the predictive values of the diagnosis than its specificity or sensitivity. Generally, in cases of FIGO stages ≥IIB, gynecologists would get the greatest benefit from tests with a high NPV for a reliable negative test result (negative is stage ≤IIA), which could change their treatment decision from conservative treatment to surgery.

For patients with FIGO stage ≤IIA, PPV is clinically the most important value for treatment planning because a reliable positive test result (positive stage ≥ stage IIB) might highly influence gynecologic oncologists to change their treatment decision to prevent unnecessary treatment after curative surgery. Therefore, a high PPV helps reduce the substantial risk of additional treatment after curative surgery by providing information regarding the presence of parametrial invasion.

Notably, the 3T multiparametric MRI positive predictive value (PPV) for parametrial invasion is 54% (29/54), which is moderate in this patient group [25]. Thus, 25/54 (46.2%) of patients were incorrectly predicted to have parametrial invasion and would prefer external beam radiotherapy to RH in cases of suspected pT2b cervical cancer to prevent unnecessary abolition of curative surgery and external beam radiotherapy would be preferable to surgery in cases of suspected pT2b cervical cancer to prevent the unnecessary rejection of curative surgery.

In this study, furthermore, compared with surgical staging, the overall accuracy of MRI stage was 69%; underdiagnosis and overdiagnosis occurred with 13-46% and 2-46% in each MRI stage, respectively (Table 5). Our results showed that MRI staging resulted in more inaccurate diagnoses, particularly for MRI stage T0 and advanced stages.

Regarding MRI stage misdiagnosis, our results demonstrated that underdiagnosis is affected by MRI tumor size and histologic cell type. Overdiagnosis is also influenced by MRI tumor size, MRI parametrial invasion, and MRI uterine corpus invasion.

The primary treatment options for early cervical cancer are surgery or chemoradiotherapy [8, 9, 14]. However, there are advantages to the use of surgery instead of radiotherapy, particularly in younger women for whom ovarian preservation might be important. From a clinical perspective, a dilemma exists regarding the reliability of MRI results to guide treatment options and design. Such reliability is particularly germane in case of early cervical cancer patients diagnosed as MRI T2b stage, notably in young women.

**Figure 2:**
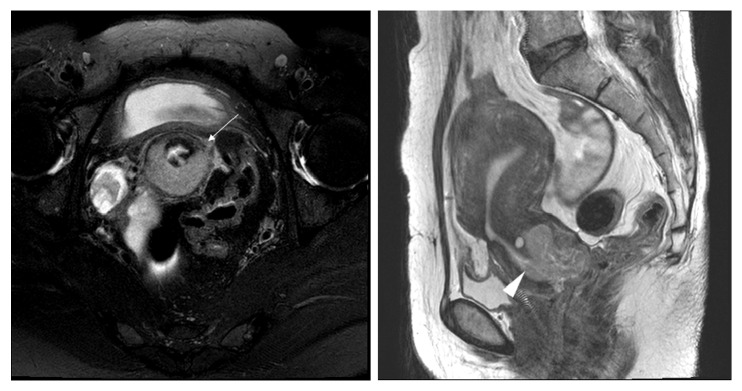
Stage IB1 cervical adenocarcinoma in a 42-year-old woman with radical hysterectomy (A) T2-weighted axial MR images show a moderated lobulated mass of uterine cervix with a focal disruption (arrow) of peripheral rim. The maximum diameter of the lesion is measured 3.6cm on the T2-weighted sagittal image. At histopathological finding, no parametrical lesion was found. MRI stage T2b (≤ 4 cm) was overdiagnosed as final pathologic stage T1b1.

**Figure 3:**
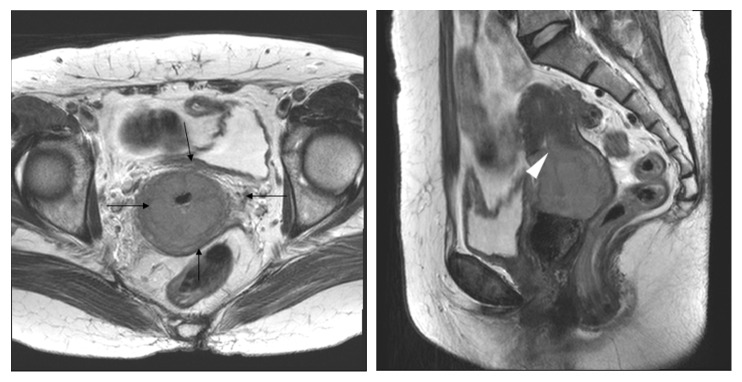
Stage IB2 squamous cervical carcinoma in a 49-year-old woman with radical hysterectomy (A) T2-weighted axial MR images show a 6cm sized lobulated concentric tumoral thickening of whole cervical stroma with an ill-defined peripheral margin (arrow) and invasion to endomyometrium(arrowhead); the maximum diameter of the lesion is measured 6cm on the T2-weighted sagittal image. At histopathological finding, no parametrial lesion was found. MRI stage T2b (> 4 cm) was overdiagnosed as final pathologic stage T1b2.

**Figure 4:**
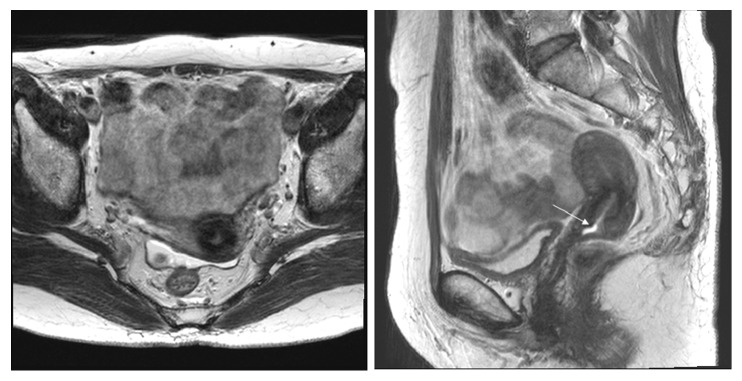
Stage IB1 squamous cervical carcinoma in a 58-year-old woman with radical hysterectomy (A) T2-weighted axial and (B) sagittal MR images show no gross cancerous lesion of uterine cervix but a tissue defect (arrow) after conization. At histopathological finding, no residual tumor in uterine cervix was found. MRI stage T0 was diagnosed as final pathologic stage T0b1.

Interestingly, 35 cases were overdiagnosed by MRI staging, whereas 25 of 35 were staged as T2b by MRI (Figures 2 and 3); these same patients were surgicopathologically staged as T2a1(3), T1b2(11) (Figure 3), or T1b1(11) (Figure 2). This error of MRI stage T2b with a comparison of MRI stage to surgicopathologic stage showed 46% in this study, whereas previous studies reported 63-67% incidence [2, 17, 25, 27]. In cases of early cervical cancer patients diagnosed as MRI T2b, the substantial likelihood of overdiagnosis should be considered.

Although MRI measured tumor size and volume seem to be strongly correlated as a predictive factor of parametrial invasion in cervical cancer, but they are still controversial [15, 18]. We have described in detail and further investigated the factors believed to affect the diagnostic inaccuracy of MRI staging, particularly regarding the predictive effect of large tumor size on misdiagnosis. Regarding MRI tumor size, when larger tumor size determined by MRI is an important factor, the possibility of overdiagnosis must be considered. In this study, we observed that tumor size > 2.9 cm is correlated highly with overdiagnosis. The larger (minimally 2.9 cm, the selected cut-off value of the ROC curve) the MRI tumor size is, the more the overdiagnoses are likely to result. In addition, we observed that, for MRI tumor size, a cut-off value of < 0.25 cm on the ROC curve correlated highly with underdiagnosis [28–30].

**Figure 5:**
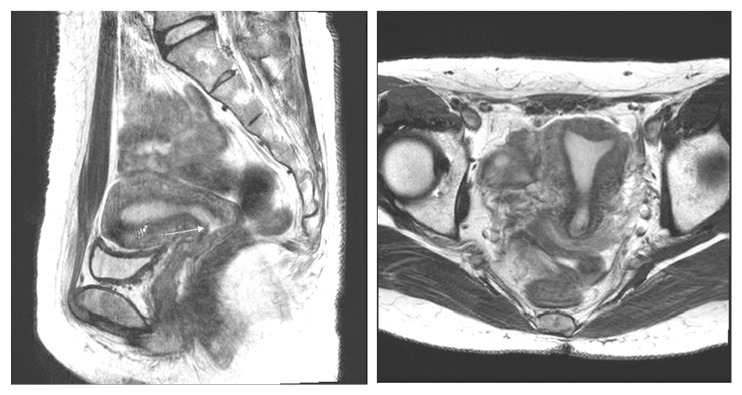
Stage IB1 squamous cervical carcinoma in a 28-year-old woman with trachelectomy (A) T2-weighted axial and (B) sagittal MR images show no gross cancerous lesion of uterine cervix but a tissue defect (arrow) after conization. At histopathological finding, 1.2 cm sized invasive squamous cell carcinoma in uterine cervix was found. MRI stage T0 was underdiagnosed as final pathologic stage T1b1.

**Figure 6:**
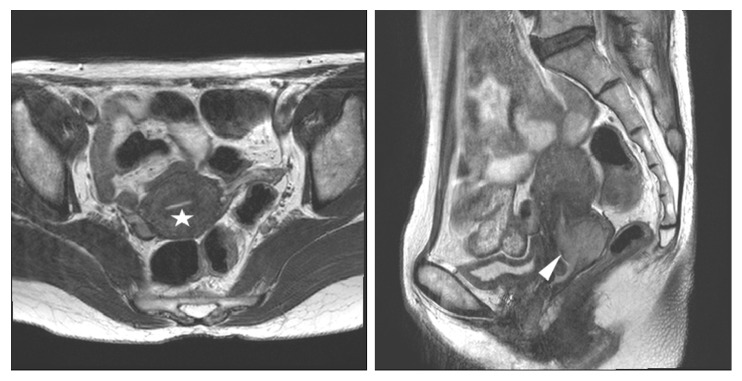
Stage IB1 squamous cervical carcinoma in a 51-year-old woman with radical hysterectomy (A) T2-weighted axial MR images show as 2.7 cm lobulated mass (star) on posterior lip of uterine cervix with no disruption of peripheral rim. The maximum diameter of the lesion is measured 2.7cm (arrowhead) on the T2-weighted sagittal image. At histopathological finding, bilateral parametrial lesion was found. MRI stage T1b (≤ 4 cm) was underdiagnosed as final pathologic stage T2b.

**Figure 7:**
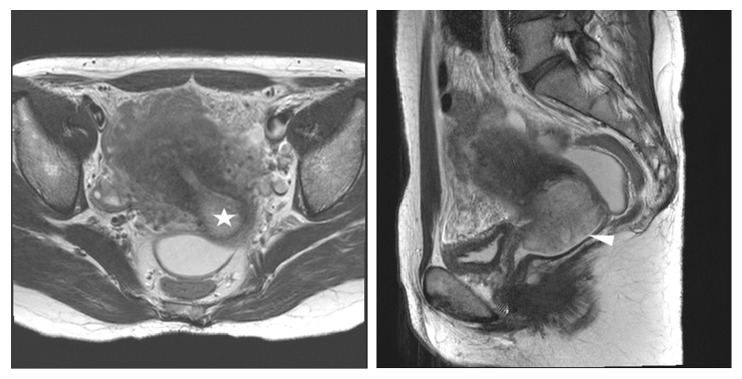
Stage IB2 cervical adenocarcinoma in a 48-year-old woman with radical hysterectomy (A) T2-weighted axial MR images show a 5 cm well defined exophytic mass (star) mainly involving right posterior exocervix with no disruption of peripheral rim. The maximum diameter of the lesion is measured 5cm (arrowhead) on the T2-weighted sagittal image. At histopathological finding, bilateral parametrial lesion was found. MRI stage T1b (> 4 cm) was underdiagnosed as final pathologic stage T2b.

Regarding MRI stage misdiagnosis, particularly underdiagnosis (Figures 5, 6, and 7), a high probability exists that the surgical treatment of patients will be affected negatively because surgical treatment in underdiagnosed cases results in inadequate surgery. Particularly for patients who wish to preserve fertility, weighing the reliability of an MRI-invisible tumor stage result before undergoing radical trachelectomy is essential (Figure 5). In previous studies, MRI was useful to detect the presence of tumor in endocervix and to guide decisions regarding fertility-sparing versus non-fertility-sparing treatment approaches [30].

This study examined additional details; for example, as pertains to underdiagnosis, specific histologic cell types such as adenocarcinoma and adenosquamous carcinoma of the cervix were significant factors. We also demonstrated that, in cases of MRI-invisible T0 stage, adenocarcinoma, and adenosquamous carcinoma histology, a high possibility existed for the presence of tumor according to the surgicopathologic results.

Several points require further discussion. First, the most contentious finding in this study, shown in Table 2, was that although MRI parametrial invasion is considered as a significant factor in predicting pathologic T2b, this study notably found that MRI showed low sensitivity and a moderate PPV for parametrial invasion [17], which is a significant predictor of overdiagnosis. Therefore, preoperative decision-making based on MRI diagnosis for early cervical cancer patients should be carefully considered, particularly in the presence of established factors that heighten the potential for misdiagnosis. Second, regarding the aforementioned underdiagnosis of MR invisible T0 stage, another crucial point is that 18 (MRI T1b stage,16; T2a,2) of 46 underdiagnosed cases warranted classifications as MRI-invisible T0 stage, because these patients are considered to have high-risk disease and accordingly may require adjuvant treatment.

Several limitations of this study should be noted. First, this study is based on a retrospective chart review, including clinical cervical cancer data. Second, most of the participants underwent conization before MRI in clinical practice [29]. Additionally, we did not assess the effects of other confounding factors, such as cervicitis, deep nabothian cysts, tunnel clusters, and endocervical hyperplasia on the accuracy of MRI interpretation. Third, although our results indicated preoperative diagnostic value of MRI staging on “young women” for whose ovarian preservation might be important and who wish to preserve fertility, our study population included approximately 45% of women who were postmenopausal women and this study was not designed to investigate young women; therefore further studies are needed to confirm this finding

## 5. Conclusions

This study suggests that preoperative decision-making for early cervical cancer patients based on MRI diagnosis should be considered carefully, especially in established factors influencing misdiagnosis. However, these findings require validation of prospective trials.

## Figures and Tables

**Table 1:** Comparison of the distribution of clinical and pathologic characteristics of the 260 women in the cohort study stratified by MRI staging.

**Clinical characteristics**	MRI T0 (n=57)	MRI T1b (n=139)	MRI T2a (n=10)	MRI T2b (n=54)	P-value^§^
**Age(y)** **∗**	47.6	49.5	47.9	52.1	0.207

**Parity** **∗**	2.1	2.2	1.9	2.3	0.773

**Operative procedure**					0.01

Open modified radical hysterectomy	2(3.5)	1(0.7)	0(0)	0(0)	

Open radical hysterectomy	9(15.8)	47(33.8)	2(20.0)	25(46.3)	

Laparoscopic modified radical hysterectomy	20(35.1)	9(6.5)	0(0)	1(1.9)	

Laparoscopic radical hysterectomy	22(38.6)	77(55.4)	8(80.0)	28(51.9)	

Laparoscopic radical trachelectomy	3(5.3)	3(2.2	0(0)	0(0)	

Robotic radical hysterectomy	1(1.8)	1(0.7)	0(0)	0(0)	

Robotic trachelectomy	0(0)	1(0.7)	0(0)	0(0)	

**Menopause **					0.004

No	37(64.9)	79(56.8)	5(50)	21(38.9)	

Yes	20(35.1))	60(43.2)	5(50)	33(61.1)	

**Cesarean section**					0.101

No	54(94.7)	122(87.8)	7(70)	46(85.2)	

Yes	3(5.3)	17(12.2)	3(30)	8(14.8)	

**History of at least one normal delivery **					0.381

No	7(12.3)	18(12.9)	5(50)	8(14.8)	

Yes	50(87.7)	121(87.1)	5(50)	46(85.2)	

**BMI **	24.1	23.9	24.3	23.8	0.948

**SCC Ag (ng/ml) ** **∗**	0.9	2.7	3.9	10.1	<0.0001

**CEA(U/ml) ** **∗**	2.7	4.9	11.1	12.3	0.098

**CA 125(U/ml) ** **∗**	18.8	15	21.4	22.8	0.441

**Histology**					0.166

Squamous	35(61.4)	94(67.6)	8(80.0)	41(75.9)	

Adenocarcinoma	21(36.8)	38(27.3)	2(20.0)	11(20.4)	

Adeno-squamous	1(1.8)	7(5)	0(0.0)	2(3.7)	

**Grade**					0.132

1	24(42.1)	20(14.4)	0(0.0)	8(14.8)	

2	19(33.3)	77(55.4)	5(50.0)	29(53.7)	

3	5(8.8)	32(23.0)	4(40.0)	13(24.1)	

Unknown	9(15.8)	10(7.2)	1(10.0)	4(7.4)	

**Biopsy type**					<0.0001

Punch biopsy	21(36.8)	106(76.3)	8(80.0)	41(77.4)	

LEEP	36(63.2)	33(23.7)	2(20.0)	12(22.6)	

**Clinical FIGO tumor stage**					<0.0001

IA	15(26.3)	0(0.0)	0(0.0)	0(0.0)	

IB1	42(73.7)	102(73.4)	4(40.0)	20(37.0)	

IB2	0(0.0)	23(16.5)	3(30.0)	26(48.1)	

IIA1	0(0.0)	6(4.3)	2(20.0)	4(7.4)	

IIA2	0(0.0)	8(5.8)	1(10.0)	4(7.4)	

**RH T category **(final pathologic_stage )					<0.0001

T1a	15(26.3)	0(0.0)	0(0.0)	0(0.0)	

T0b1	16(28.1)	3(2.2)	0(0.0)	0(0.0)	

T1b1	26(45.6)	96(66.9)	6(60.0)	11(20.4)	

T1b2	0(0.0)	25(18.0)	1(10.0)	11(20.4)	

T2a1	0(0.0)	1(0.7)	0(0.0)	3(5.6)	

T2a2	0(0.0)	1(0.7)	1(10.0)	0(0.0)	

T2b	0(0.0)	16(11.5)	2(20.0)	29(53.7)	

**Pathologic Tumor size **	0.601	2.93	4.24	4.68	<0.0001

**Pathologic tumor size **					<0.0001

0-≤1	43(75.4)	19(13.7)	0(0.0)	0(0.0)	

1-≤2	10(17.5)	21(15.1)	0(0.0)	2(3.7)	

2-≤3	2(3.5)	46(33.1)	2(20.0)	9(16.7)	

3-≤4	2(3.5)	21(15.1)	4(40.0)	11(20.4)	

4-≤5	0(0.0)	18(12.9)	3(30.0)	14(25.9)	

5-≤6	0(0.0)	13(9.4)	0(0.0)	7(13.0)	

6-≤7	0(0.0)	1(0.7)	0(0.0)	7(13.0)	

>7	0(0.0)	0(0.0)	1(10.0)	4(7.4)	

**LVSI**					<0.0001

Yes	4(7.0)	53(38.1)	7(70.0)	36(66.7)	

No	53(93.0)	86(61.9)	3(30.0)	18(33.3)	

**Pathologic deep stromal invasion**					<0.0001

Inner 1/3	48(84.2)	27(19.4)	2(20.0)	0(0.0)	

Middle 1/3	6(10.5)	44(31.7)	3(30.0)	2(3.7)	

Outer 1/3	3(5.3)	68(48.9)	5(50.0)	52(96.3)	

**Pathologic parametrial invasion**					<0.0001

No	57(100.0)	124(89.2)	8(80.0)	25(46.3)	

Yes	0(0.0)	15(10.8)	2(20.0)	29(53.7)	

**Pathologic parametrial invasion laterality**					<0.0001

Negative	57(100.0)	124(89.2)	8(80.0)	22(40.7)	

Unilateral	0(0.0)	8(5.8)	1(10.0)	14(25.9)	

Bilateral	0(0.0)	7(5.0)	1(10.0)	18(33.3)	

**Pathologic pelvic LN involvement**					<0.0001

Negative	55(96.5)	115(82.7)	6(60.0)	30(55.6)	

Positive	2(3.5)	24(17.3)	4(40.0)	24(44.4)	

**Pathologic para-aortic LN involvement**					0.001

Negative	51(89.5)	120(86.3)	9(90.0)	45(83.3)	

Positive	0(0.0)	10(7.2)	1(10.0)	8(14.8)	

Not done	6(10.5)	9(6.5)	0(0.0)	1(1.9)	

**Pathological uterine involvement**					

No	56(98.2)	125(89.9)	8(80.0)	26(48.1)	

Yes	1(1.8)	14(10.1)	2(20.0)	28(51.9)	

**MRI tumor size** **∗**	0.00	2.89	4.06	4.61	<0.0001

**MRI pelvic LN involvement**					

Negative	57(100.0)	128(92.1)	7(70.0)	36(66.7)	

Positive	0(0.0)	11(7.9)	3(30.0)	18(33.3)	

**MRI para-aortic LN involvement**					0.46

Negative	57(100.0)	137(98.6)	10(100.0)	53(98.1)	

Positive	0(0.0)	2(1.4)	0(0.0)	1(1.9)	

**MRI uterine corpus invasion**					

No	57(100.0)	127(91.4)	8(80.0)	27(50.0)	

Yes	0(0.0)	12(8.6)	2(20.0)	27(50.0)	

**MRI deep stromal ** ** invasion**					

No invasion	57(100.0)	2(1.4)	0(0.0)	0(0.0)	

Partial invasion	0(0.0)	79(56.8)	6(60.0)	3(5.6)	

Complete invasion	0(0.0)	58(41.7)	4(40.0)	51(94.4)	

The data are presented as the number (%) or the mean ± SD.

FIGO, International Federation of Gynecology and Obstetrics; RH, radical hysterectomy; LVSI, lymphovascular stromal invasion; LN, lymph node; CA 125, carbohydrate antigen 125; CEA, carcinoembryonic antigen; CA 19-9, carbohydrate antigen 19-9; MRI, magnetic resonance imaging.

**Table 2:** Adjusted odds ratios with 95% confidence intervals (CI) and associated P values from the logistic regression predicting pathologic category T2b in the 203 study cohort patients.

**Clinical characteristics**	**No. of patients**	**No. of events** **(**%** of patients)**	**Univariable analysis**		**Multivariable analysis**	
			**UOR (95**%** CI)**	***P***	** AOR (95**%** CI)**	***p***
**Age(y)**	203	47(23.2)	1.05(1.02-1.09)	0.001	1.05(1.01-1.09)	0.009

**Parity**	203	47(23.2)	0.99(0.76-1.31)	0.992		

**BMI**	203	47(23.2)	1.04(0.95-1.13)	0.424		

**Cesarean section**						

No	175	42(24)	1	0.476		

Yes	28	5(17.9)	0.69(0.25-1.92)			

**History of at least one normal delivery**	203			0.401		

No	31	9(29)	1			

Yes	172	38(22.1)	0.69(0.29-1.63)			

**Menopause**	203			0.007		

No	105	16(15.2)	1			

Yes	98	31(31.6)	2.57(1.30-5.09)			

**Histology**				0.374		

Squamous	143	31(21.7)	1			

Adenocarcinoma	51	15(29.4)	1.51(0.73-3.09)			

Adenosquamous	9	1(11.1)	0.45(0.05-3.75)			

**Grade**				0.45		

1	28	7(25)	1			

2	111	24(21.6)	0.83(0.32-2.18)			

3	49	10(20.4)	0.77(0.26-2.31)			

Unknown	15	6(40)	2.00(0.52-7.65)			

**SCC ag(ng/mL)**	203	47(23.2)	1.04(1.01-1.06)	0.012		

**MRI T stage**				<0.0001		

T1b	103	16(11.5)	1			

T2a	10	2(20)	1.92(0.38-9.86)			

T2b	54	29(53.7)	8.92(4.23-18.82)			

**Clinical FIGO stage**				<0.0001		

IB1	126	20(15.8)	1			

IB2	52	19(36.5)	7.41(3.04-16.12)			

IIA1	12	3(25)	2.67(0.65-11.03)			

IIA2	13	5(38.5)	5.00(1.44-17.41)			

**MRI tumor size **	203	47(23.2)	1.84(1.46-2.32)	<0.0001		

**MRI pelvic LN involvement**				0.013		

Negative	171	34(19.9)	1			

Positive	32	13(40.6)	2.76(1.24-6.13)			

**MRI parametrial invasion**				<0.0001		0.002

No	146	18(12.3)	1		1	

Yes	57	29(50.9)	7.37(3.59-15.08)		3.77(1.62-8.79)	

**MRI parametrial invasion**				<0.0001		

Negative	146	17(11.6)	1			

Unilateral	46	21(45.7)	6.37(2.95-13.76)			

Bilateral	11	9(81.8)	34.15(6.80-171.43)			

**MRI vaginal involvement**				0.60		

No	193	44(22.8)	1			

Yes	10	3(30)	1.45(0.36-5.85)			

**MRI uterine corpus invasion**				<0.0001		<0.0001

No	162	19(11.7)	1		1	

Yes	41	28(68.3)	16.21(7.19-36.57)		9.99(4.11-24.32)	

**Table 3:** Univariable and multivariable analysis of factors associated with diagnostic inaccuracy of preoperative MRI staging.

	**Under-diagnosis**				**Over-diagnosis**			
	Univariable		Multivariable		Univariable		Multivariable	
	**UOR ** **95**%** CI**	**Over- ** **all P**	**AOR ** **95**%** CI**	**Over- ** **all P**	**UOR ** **95**%** CI**	**Over-all P**	**AOR ** **95**%** CI**	**Over- ** **all P**
**Age(y)**	1.02 (0.99-1.05)	0.254			0.99 (0.95-1.02)	0.409		

**Menopause**		0.605				0.394		

No	1				1			

Yes	1.19 (0.62-2.27)				1.37 (0.66-2.83)			

**Parity**	1.08 (0.83-1.39)	0.584			1.13 (0.82-1.55)	0.449		

**History of at least one normal delivery**		0.751				0.314		

No	1				1			

Yes	0.86 (0.35-2.15)				0.62 (0.43-1.58)			

**Histology**		0.521				0.159		

Squamous	1				1			

Adenocarcinoma	2.11 (1.07-4.16)				0.25 (0.074-0.87)			

Adeno-squamous	1.72 (0.33-9.05)				1.38 (0.27-7.17)			

		0.031		0.034		0.055		0.064

Squamous	1		1		1		1	

Adenocarcinoma or adenosquamos	2.07 (1.07-3.99)		2.07 (1.06-4.07)		0.38 (0.14-1.02)		0.34 (0.11-1.07)	

**SCC ag(ng/mL)**	0.96 (0.89-1.03)	0.257			1.01 (0.99-1.04)	0.341		

**MRI tumor size**	0.76 (0.63-0.92)	0.004	0.76 (0.63-0.92)	0.005	1.46 (1.18-1.81)	0.001	1.51 (1.06-2.16)	0.023

**MRI parametrial invasion**		0.029				<0.0001		<0.0001

No	1				1		1	

Yes	0.11 (0.14-0.79)				11.94 (5.21-27.35)		73.73 (8.89-611.38)	

**MRI uterine corpus invasion**		0.347				0.363		0.003

No	1				1		1	

Yes	1.49 (0.65-3.49)				1.54 (0.61-3.90)		0.13 (0.03-0.49)	

**MRI deep stromal invasion**		0.840				0.006		0.051

No invasion & partial invasion	1				1		1	

Complete invasion	1.07 (0.55-2.07)				2.92 (1.37-6.23)		0.19 (0.01-1.01)	

**Table 4:** Comparison between final pathologic stage and MRI radiologic stage.

MRI radiologic stage	Final pathologic stage							
	T2b (%)	T2a2(%)	T2a1(%)	T1b2(%)	T1b1(%)	T0b1*∗* (%)	T1a (%)	Total
T2b	29(61.7)	0(0)	3(75)	11(29.7)	11(8.1)	0	0	54

T2a	2(4.3)	1(50)	0(0)	1(2.7)	6(4.4)	0	0	10

T1b	16(34)	1(50)	1(25)	25(67.6)	93(68.4)	3(15.8)	0	139

T0	0	0	0	0	26(19.1)	16(84.2)	15(100)	57

	47	2	4	37	136	19	15	260

*∗* After a positive biopsy for infiltrating carcinoma, no tumor was found in the cervix in the surgical specimen.

**Table 5:** Incidence of staging errors.

MRI Stage	Errors and incidence with a comparison of MRI stage to surgico-pathologic stage
T0(57 patients)	

Understaged (26:46%)	T1b1: Tumor confined to the cervix or microscopic lesion greater than T1a1/a2, in greatest dimension (26)

T1b (139 patients)	

Understaged (18:13%)	T2a1: Cervical carcinoma invades beyond uterus but not to pelvic wall or to lower third of vagina, ≤4 cm in greatest dimension (1)
	T2a2: Cervical carcinoma invades beyond uterus but not to pelvic wall or to lower third of vagina > 4.0 cm in greatest dimension (1)
	T2b: Tumor with parametrial invasion (16)

Overstaged (3:2%)	T0b1: After a positive biopsy for infiltrating carcinoma, no tumor was found in the cervix in the surgical specimen (3)

T2a (10 patients)	

Understaged (2:20%)	T2b: Tumor with parametrial invasion (2)

Overstaged (7:70%)	T1b1: Tumor limited to cervix or microscopic lesion greater than T1a1/a2, ≤ 4.0 cm in greatest dimension (6)
	T1b2: Tumor limited to cervix or microscopic lesion greater than T1a1/a2, > 4.0 cm in great dimension (1)

T2b (54 patients)	

Overstaged (25:46%)	T1b1: Tumor limited to cervix, ≤ 4.0 cm or less in greatest dimension (11)
	T1b2: Tumor limited to cervix, > 4.0 cm in great dimension (11)
	T2a1: Cervical carcinoma invades beyond uterus but not to pelvic wall or to lower third of vagina, ≤4 cm in greatest dimension (3)

**Table 6:** Clinical performance of the cut-off lesion size measured on MRI to predict the effect of large tumor size for misdiagnosis (overdiagnosis and underdiagnosis) with optimal accuracy.

		AUC (95% CI)	Threshold(cm)	Sensitivity (%)	Specificity (%)	Accuracy (%)
Overdiagnosis	MRI tumor size	0.68 (0.60-0.77)	> 2.9	80	53	58

Underdiagnosis	MRI tumor size	0.64 (0.54-0.75)	< 0.25	56.5	83	77

## Data Availability

The data used to support the findings of this study are available from the corresponding author upon request.

## Supplementary Materials

Supplementary Materials Supplementary Table 1: three-tesla multiparametric MRI sequence parameters. Supplementary Table 2: American Joint Committee on Cancer (AJCC)-Tumor-Node-Metastases(TNM), International Federation of Gynecology Obstetrics(FIGO), and MRI surgical staging systems for carcinoma of the cervix.
