# Supplementary material for: Early Cervical Cancer: Predictive Relevance of Preoperative 3-Tesla Multiparametric Magnetic Resonance Imaging
Source: Int J Surg Oncol. 2018 Aug 1;2018:9120753. doi: 10.1155/2018/9120753 (PMC6092969; doi:10.1155/2018/9120753)
Supplement: Supplementary Materials — Supplementary Table 1: three-tesla multiparametric MRI sequence parameters. Supplementary Table 2: American Joint Committee on Cancer (AJCC)-Tumor-Node-Metastases(TNM), International Federation of Gynecology Obstetrics(FIGO), and MRI surgical staging systems for carcinoma of the cervix. [file 9120753.f1.docx]

Supplementary Table 1. Three-tesla multiparametric MRI sequence parameters

| Imaging parameters | T1W  axial | T2W axial/coronal/sagittal | T1W axial  precontrast/  postcontrast | DWI |
| --- | --- | --- | --- | --- |
| Sequence type | TSE | TSE | TSE | SE-EPI |
| TR(ms) | 535 | 4000 | 729 | 5650 |
| TE(ms) | 8.8 | 90 | 8.8 | 64 |
| Slice thickness/gap(mm) | 4/0.4mm | 4/0.4mm | 4/0.4mm | 4/0.4mm |
| Number of slices per station | 30 | 30 | 30 | 30 |
| FOV(mm) | 200x200 | 200x200 | 200x200 | 250x250 |
| Matrix | 400x284 | 284x284 | 400x285 | 124x124 |
| Bandwidths(Hz) | 218.4 | 229.9 | 218.7 | 24.7 |
| Scans/ station(s) | 120s | 160s | 280s | 163s |
| Flip angle (°) | 90 | 90 | 90 | 90 |
| Number of signal averages | 2 | 2 | 2 | 4 |
| b-values(s/mm^2^) |  |  |  | 0,800 |

Abbreviations: T2W = T2-weighted; DWI =diffusion-weighted MR imaging; DCE =dynamic contrast enhanced MR imaging; FOV=field of view; TSE= turbo-spin echo; SE-EPI= spin-echo echo-planar imaging; GRE= gradient echo sequence; FLASH= fast low angle shot; TR= Time to Repetition; TE= Time to Echo; TI= time to inversion; ms= milliseconds

Supplementary Table 2. American Joint Committee on Cancer (AJCC)-Tumor-Node-Metastases(TNM), International Federation of Gynecology Obstetrics(FIGO) and MRI surgical staging systems for carcinoma of the cervix

| Pathologic or MRI TNM  categories | FIGO  stages | Surgical-Pathologic Findings | MRI findings |
| --- | --- | --- | --- |
| TX |  | Primary tumor cannot be assessed |  |
| T0 |  | No evidence of primary tumor | Not visible |
| T1a1,2 | IA1,2 | Invasive carcinoma diagnosed only by microscopy, stromal invasion with a maximum depth 5.0 mm measured from the base of the epithelium and a horizontal spread of 7.0mm or less. vascular space involvement, venous or lymphatic, does not affect classification | No tumor visible |
| T1b | IB | Clinically visible lesion confined to the cervix or microscopic lesion greater than T1a/A2 | Tumor identified but an intact cervical stromal ring surrounding the tumor. |
| T1b1 | IB1 | Clinically visible lesion 4.0 cm or less in greatest dimension | Tumor identified but an intact cervical stromal ring surrounding the tumor. |
| T1b2 | IB2 | Clinically visible lesion more than 4.0cm in great dimension |  |
| T2 | II | Cervical carcinoma invades beyond uterus but not to pelvic wall or to lower third of vagina | Proximity of the margin of the cervical tumor to vagina associated with loss of the low SI vaginal wall but no extension of tumor beyond. |
| T2a | IIA | Tumor without parametrial invasion | Loss of integrity of the low-signal intensity vaginal wall (upper two-thirds) |
| T2a1 | IIA1 | Clinically visible lesion 4.0cm or less in greatest dimension |  |
| T2a2 | IIA2 | Clinically visible lesion more than 4.0cm in greatest dimension |  |
| T2b | IIB | Tumor with parametrial invasion | Disrupted low-signal intensity ring of the cervix with tumor extending into the parametrium |
| T3 | III | Tumor extension to pelvic wall and/or involves lower third of vagina and /or causes hydronephrosis or nonfunctioning kidney |  |
| T3a | IIIA | Tumor involves lower third of vagina, no extension to pelvic wall | Tumor extending into the lower third of vagina |
| T3b | IIIB | Tumor extends to pelvic wall and/or causes hydronephrosis or nonfunctioning kidney | Tumor extending to pelvis muscles or dilated ureter. |
| T4 | IVA | Tumor invades mucosa of bladder or rectum, and/or extends beyond true pelvis (bullous edema is not sufficient to classify a tumor as T4) | Loss of perivesical and perirectal fat planes, tumor extending to the bladder or rectum. |
|  | IVB | Distant metastasis (including peritoneal spread, involvement of supraclavicular, mediastinal, or paraaortic lymph nodes, lung, liver, bone) | Tumor demonstrated outside the true pelvis |
